# Pre-emptive genomic surveillance of emerging ebolaviruses

**DOI:** 10.2807/1560-7917.ES.2020.25.3.1900765

**Published:** 2020-01-23

**Authors:** Ignacio Postigo-Hidalgo, Carlo Fischer, Andres Moreira-Soto, Patricia Tscheak, Michael Nagel, Markus Eickmann, Jan Felix Drexler

**Affiliations:** 1 Charité—Universitätsmedizin Berlin, Corporate Member of Freie Universität Berlin, Humboldt-Universität zu Berlin and Berlin Institute of Health, Institute of Virology, Berlin, Germany; 2 Deutsche Gesellschaft für Internationale Zusammenarbeit (GIZ) GmbH, Bonn, Germany; 3 German Centre for Infection Research (DZIF), partner site Giessen - Marburg – Langen, Germany; 4 Institute of Virology, Philipps-Universität Marburg, Marburg, Germany; 5 German Centre for Infection Research (DZIF), associated partner Charité - Universitätsmedizin Berlin, Berlin, Germany

**Keywords:** Ebolavirus, genomic surveillance, PCR, nested PCR

## Abstract

Genomic surveillance during ebolavirus outbreaks to elucidate transmission chains and develop diagnostic tests is delayed by the laborious development of variant-specific laboratory assays. We developed a new protocol combining 31 parallel PCR assays with Illumina/MinION-based sequencing, allowing generic ebolavirus genomic surveillance, validated using cell culture-derived Ebola, Reston, Sudan and Taï Forest virus at concentrations compatible with patient viral loads. Our approach enables pre-emptive genomic surveillance of ongoing and future ebolavirus outbreaks irrespective of variant divergence.

From 2012 to 2017, the Democratic Republic of the Congo (DRC) experienced three small isolated ebolavirus outbreaks, two of them caused by different variants from the species *Zaire ebolavirus* and one by a variant from the species *Bundibugyo ebolavirus*. Starting in 2018 in DRC, a large ongoing Ebola virus (EBOV) outbreak caused by different *Zaire ebolavirus* variants became the second-largest outbreak ever recorded and was declared a Public Health Emergency of International Concern, a classification only conferred to five other epidemics in history by the World Health Organization [1].

## Delayed availability of protocols for genomic characterisation 

**Figure 1:**
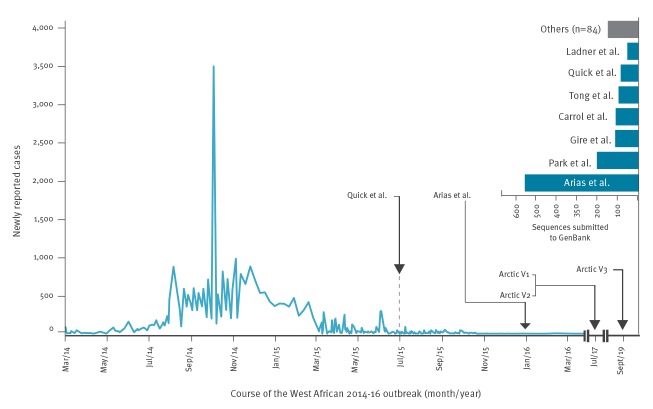
Delayed availability of sequencing protocols for Ebola viruses, West Africa, 2014–2019 (n = 28,646 cases)

During the large West African EBOV outbreak in 2014-16, primer schemes enabling PCR-based full genome characterisation were not available until late in the course of the epidemic (Figure 1), partly because of the time necessary to develop and validate suitable assays in the laboratory. In addition, the ability to transfer those protocols between groups remained unclear because most viral genomes produced from that outbreak were obtained by different groups using different protocols containing complex multiplexed PCR reactions and optimised for distinct deep sequencing platforms (Figure 1). Notably, deep sequencing platforms may not produce sufficient numbers of viral reads from some clinical samples without genomic pre-amplification or target enrichment [2], mainly because the amounts of viral nucleic acid are small compared with the host’s own nucleic acid in clinical samples [3]. This can result in a lack of genomic coverage to conduct robust genomic surveillance, which is necessary for diagnostic test development, elucidation of transmission chains and monitoring of mutations potentially affecting ebolavirus pathogenesis or transmissibility [4,5]. Advanced diagnostics for emerging viruses such as EBOV are thus urgently needed to support outbreak response in Europe and beyond [6].

EBOV: Ebola virus.

Blue line: newly reported EBOV cases during the West African EBOV outbreak in 2014–16 (assembled using case data provided by WHO Situation Reports - https://www.who.int/csr/disease/ebola/situation-reports/archive/en/) and publication of primer schemes for full genome sequencing. Horizontal bars: number of sequences submitted to GenBank by different authors since the beginning of the outbreak [7,8,16-19]. Arrows: date of release of primer schemes for EBOV full genome characterisation during and after the outbreak (Artic V1 and V2 release dates were not explicitly specified, see shared arrow).

The assay from Quick et al. comprises two primer schemes of 11 and 19 primer pairs and was validated for use on the MinION platform. Notably, the authors made their assay available in July 2015, almost 1 year ahead of publication [20]. The assay from Arias et al. [8] relies on a proprietary workflow termed Ion Ampliseq and comprises two sets of 72 and 73 primer pairs each, which are to be amplified in two separate multiplexed reactions and sequenced on an Ion Torrent platform; the Artic schemes are part of a set of online resources for EBOV MinION-based sequencing. The first version has been available since 2017 in github (https://github.com/artic-network/primer-schemes), however optimisation of the protocol is still ongoing, given the release of the most recent version in 2019. The current version of the assay comprises 61 primer pairs to be multiplexed in two PCR reactions containing 30 and 31 primer pairs each.

To address this need, we developed a generic PCR-/deep sequencing-based protocol that is suitable for pre-emptive ebolavirus genomic surveillance irrespective of variant divergence.

## Development of a generic ebolavirus PCR protocol

**Figure 2:**
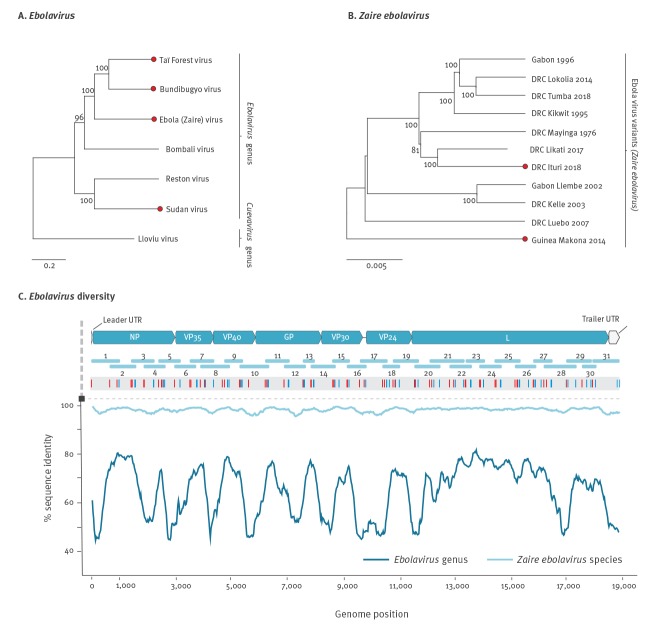
Genomic diversity and generic ebolavirus assay design

The genus *Ebolavirus* comprises six species, of which four contain known human pathogens, namely Ebola (species *Zaire ebolavirus*), Sudan (species *Sudan ebolavirus*), Bundibugyo (species *Bundibugyo* *ebolavirus*) and Taï Forest (species *Taï Forest* *ebolavirus*) virus (Figure 2A). All ebolaviruses share a non-segmented, linear negative-sense RNA genome of ca 18.9 kb. The genome comprises seven genes that are flanked by untranslated regions (UTR). To date, 70% of ebolavirus outbreaks have been caused by members of the species *Zaire ebolavirus, *however, in each outbreak, new variants arose (Figure 2B). EBOV-specific protocols are available and feasible given that the overall genomic identity is around 98% (Figure 2C). Published PCR-based assays capable of amplifying members of different EBOV variants comprise 30–60 primer pairs, such as the protocol from Quick et al. [7] or the Artic V3 protocol available only online (https://artic.network/ebov/), both designed for MinION-based downstream sequencing. Another protocol from Arias et al. [8] contains up to 140 primer pairs amplifying small genomic fragments with downstream Ion Torrent-based sequencing. These protocols use single-round conventional PCR in a highly multiplexed approach to reduce the number of reactions [8,9]. Beyond EBOV protocols, the development of a genus-wide protocol is challenging given that the average sequence identity across the genome of all known variants comprised in the different species forming the *Ebolavirus* genus is only around 62% (Figure 2C) [10].

UTR: untranslated region.

A. Phylogenetic diversity of the *Ebolavirus* genus. Red circles: viruses from species known to cause disease in humans. MEGA (https://www.megasoftware.net/) was used to infer the evolutionary history using the neighbour-joining method and the maximum composite likelihood method, full deletion of gaps and ambiguous sites was chosen; number at nodes represent statistical support of grouping from 1,000 bootstrap replicates;

B. Phylogenetic diversity of the *Zaire ebolavirus* species. Strains are given by country and outbreak year. Red circles: variants responsible for the largest EBOV outbreaks to date. The tree was calculated with the same methods as in panel A.

C. Sequence identity plot comparing diversity within *Zaire ebolavirus* variants and within the *Ebolavirus* genus; and generic ebolavirus assay scheme. Header: scheme of ebolavirus genome organisation; numbered horizontal lines in light blue: position of the amplicons of the first round; vertical lines: the specific position of the first and second round primers; red lines: forward primers; blue lines: reverse primers. The identity plot was calculated in SSE (http://www.virus-evolution.org/Downloads/Software/), using an alignment of representative sequences from members of each ebolavirus species or members of the species *Zaire ebolavirus* (as in panels A and B); a fragment length of 500 nt. and an increment between fragments of 25 nt were selected.

GenBank accession numbers from the taxa used in A and B can be found in the Supplement.

To address this, we retrieved and aligned all available ebolavirus full or partial genomes from GenBank (accessed on 22 June 2019) and removed identical sequences (n = 2,368 sequences in the final dataset). We included the most closely related Lloviu virus, retrieved from Spanish bats and classified in the *Ebolavirus* sister genus *Cuevavirus* in the alignment, to allow for the identification of highly conserved sites across the genomes. This was done because mutations are not spread evenly across viral genomes and conserved areas exist because of functional domains or RNA secondary structures limiting mutations in a given genomic region. With this approach, the assay can be used long-term and predictably for divergent ebolaviruses, as shown for other viruses before [11,12]. Following several rounds of primer design and experimental validation of assay sensitivity on quantified EBOV Makona RNA, the final protocol comprised two PCR rounds comprising 31 overlapping assays per run, with an average product size of around 900 bp and at least 50 nt overlap (Figure 2C, Supplementary Tables S1 and S2, Supplementary Figures S1 and S2). In those genomic areas where it was not possible to identify conserved regions across the ebolavirus species, species-specific primers were developed and multiplexed (Supplementary Table S1 and S2, Supplement: Generic ebolavirus assay bench protocol, Supplementary Figure S3).

## Validation of the generic ebolavirus PCR protocol for genomic sequencing

To validate the generic ebolavirus PCR protocol, we used cell culture-derived full-length virus RNA representing members of four different *Ebolavirus* species, namely *Zaire*, *Reston*, *Sudan* and *Taï Forest ebolavirus* (Supplementary methods).

**Figure 3:**
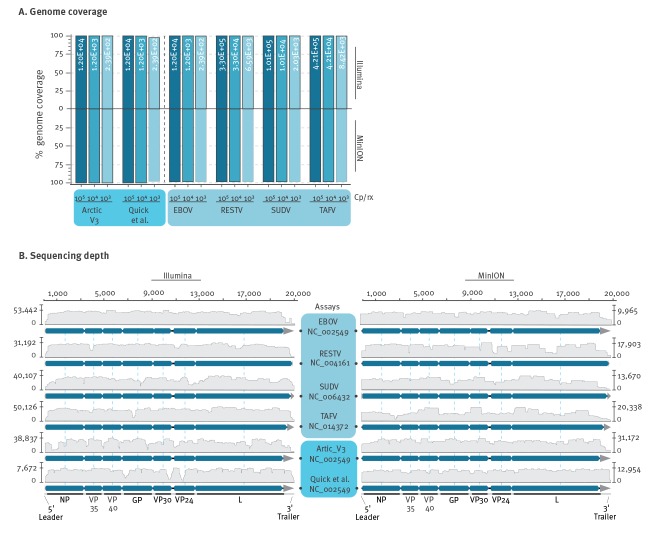
Genome coverage and sequencing depth of ebolavirus assays

EBOV: Ebola virus; RESTV: Reston virus, SUDV: Sudan virus; TAFV: Taï Forest virus.

We were able to recover the complete genome of all viruses from reactions containing on average 10^5^, 10^4^ and 10^3^ viral copies per reaction both in Illumina- and in MinION-based sequencing (Figure 3A). Note that classical Sanger sequencing would also be possible given the overlap between amplicons. The median viral load reported from patients during the large West-African epidemic in 2014–16 corresponded to ca 10^3^ viral copies/µL of plasma (interquartile range: 10^2^–10^6^) [13]. Because we successfully typed ebolavirus genomes from comparable amounts of cell culture supernatant, the sensitivity of our assay is likely to be compatible with viral loads in most patients, including chiefly symptomatic patients who have relatively higher viral loads than asymptomatic cases [14].

Reads from the first and second round were mapped together against reference sequences of each ebolavirus species.

A. Percentage of genome coverage achieved by each assay with an average of 10^5^, 10^4^ and 10^3^ copies per reaction. The exact number of copies per reaction is indicated in white text at the top of the bar.

B. Sequencing depth by nucleotide position achieved by our assay for each ebolavirus species at an average of 10^3^ copies per reaction and by the Artic V3 and Quick et al. protocols for the same EBOV specimen used in the generic protocol. Identical PCR products were used for Illumina- and MinION-specific library preparation and downstream sequencing platforms. Mapping only the first round yielded similar results in high-titred specimens (≥ 10^4^ copies/reaction) (data not shown).

In terms of sequencing depth, reactions containing on average 10^3^ viral copies yielded around 2,000–10,000 reads/nt for all ebolavirus tested (Figure 3B). At ca 10-fold lower amounts of viral copies per reaction, we were still able to achieve a coverage ranging from 55 to 95 per cent of the analysed ebolavirus genomes (Supplementary Figure S4). In sum, we achieved high genomic coverage and sequencing depth for divergent ebolaviruses that was comparable to that achieved by EBOV-specific assays [8,9]. Even if the generic protocol may be less sensitive than variant-specific approaches and fail to retrieve full genomes at lower virus concentrations such as in oligosymptomatic patients or in patients sampled during recovery, sufficient genomic information will be obtainable to design diagnostic protocols, taxonomically classify the ebolavirus variants causing the outbreak and complete full genomes by designing variant-specific primers bridging the sequence islets retrieved by the generic protocol.

## Limitations

A limitation of our study is that our virus panel did not include members of the species *Bundibugyo* and *Bombali ebolavirus*. Nonetheless, the validation with Taï Forest virus may represent the genetically closely related Bundibugyo virus; Bombali viruses are bat viruses not known to be pathogenic for humans [15]. Another limitation is the usage of modified bases such as inosines that increases the cost of primers. However, usage of such bases was required to reduce primer degeneracy. Another limitation is that we opted for a nested PCR protocol, which increases pipetting complexity and the risk of amplicon contamination. Standard laboratory precautions to avoid contamination must therefore be applied. Finally, although usage of both rounds is recommended, using the first round protocol only may provide sufficient results in cases of high viral load such as in symptomatic patients [14]. It is important to note that this protocol is designed for genomic characterisation of ebolaviruses, and is neither intended nor validated for diagnostic purposes.

## Conclusions

Reactive set-up of protocols for genomic surveillance in outbreak settings takes months of laboratory development and dissemination within the scientific community, hindering timely outbreak response. The generic ebolavirus PCR protocol presented here is designed to circumvent these limitations and contribute to genomic surveillance of current and emerging ebolavirus variants. This will be useful both in resource-limited areas, exemplified by the current outbreak affecting the DRC, and in laboratories testing healthcare workers and travellers from outbreak areas.

## Supplementary Data

693000 Supplement
