# Supplementary material for: Pre-emptive genomic surveillance of emerging ebolaviruses
Source: Euro Surveill. 2020 Jan 23;25(3):1900765. doi: 10.2807/1560-7917.ES.2020.25.3.1900765 (PMC6988270; doi:10.2807/1560-7917.ES.2020.25.3.1900765)
Supplement: Supplement [file 19-00765_DREXLER_Supplement.pdf]

This supplementary material is hosted by *Eurosurveillance* as supporting information alongside the article "Pre-emptive genomic surveillance of emerging ebolaviruses", on behalf of the authors, who remain responsible for the accuracy and appropriateness of the content. The same standards for ethics, copyright, attributions and permissions as for the article apply. Supplements are not edited by *Eurosurveillance* and the journal is not responsible for the maintenance of any links or email addresses provided therein.

Supplementary methods

Important note: This protocol is designed for genomic characterization of ebolavirus variants, and is neither intended, nor validated for diagnostic purposes.

RNA Extraction and downstream analysis

We mixed 140 µL of cell-culture supernatant from each ebolavirus species with 560 µL of AVL for inactivation and 560 µL of ethanol. From 200 µL of the previous mix, we extracted the RNA and eluted it in 60 µL of elution buffer (10 mM Tris-Cl, pH 8.5), using a QIAamp Viral RNA kit (Qiagen, Hilden, Germany) following the manufacturer's instructions. We quantified those viruses using the Filovirus real-time RT-PCR typing kit (Altona Diagnostics, Hamburg, Germany) and species-specific cRNA *in-vitro* transcripts. We generated cDNA for downstream PCR testing from the eluted RNA using SSIII reverse transcriptase (Thermofisher, Darmstadt, Germany) and random-hexamer primers following the manufacturer's specifications. After amplification, we prepared amplicon pools for each ebolavirus species and constructed DNA libraries for Illumina MiSeq (V2 chemistry) using the Kapa HyperPrep DNA library preparation kit (Roche, Penzberg, Germany) and for MinION (Oxford Nanopore, Oxford, UK) using a Ligation Sequencing Kit 1D.

Dataset creation and alignments

All datasets were created and handled in Geneious Prime (https://www.geneious.com). Sequences were aligned using MAFFT [1] implemented as a plugin in Geneious.

GenBank accession numbers from **Figure 2A**, are as follows: AF086833.2, FJ217162.1, FJ217161.1, MF319185.1, AF522874.1, AY729654.1, JF828358.1

GenBank accession numbers from **Figure 2B**, are as follows: KC242784, KC242791, KC242792, KC242796, KC242799, KC242800, KF113529, KJ660346, KM519951, MH481611, MH733481.

Mapping of the sequences

Sequences were mapped to reference sequences of each ebolavirus species using Geneious Mapper implemented in Geneious Prime, using reads from 1st and 2nd round and choosing the options "Trim Sequences – Trim Primers" and "Fine tuning – two iterations". Reference sequences per species:

- *Zaire ebolavirus* – NC_002549
- *Reston ebolavirus* – NC_004161
- *Sudan ebolavirus* – NC_006432
- *Taï Forest ebolavirus* – NC_014372

Ebolavirus nomenclature used in this paper

All references to ebolaviruses and the underlying taxonomical concepts are done according to Kuhn, 2017 [2].

Taxonomical concepts:

- Genus: *Ebolavirus*
- Species: *Zaire ebolavirus, Reston ebolavirus, Taï Forest ebolavirus, Sudan ebolavirus, Bundibugyo ebolavirus, Bombali ebolavirus*.

Members of the taxonomical concepts (objects):

- Member(s) of genus: ebolavirus(es)
- Member(s) of species: Ebola virus (former Zaire ebolavirus), Reston virus, Taï Forest virus, Sudan virus, Bundibugyo virus, Bombali virus.
    - Abbreviation of members: EBOV (former ZEBOV), RESTV, SUDV, TAFV, BDBV, BOMV.

The term variant(s) refer to the taxonomical concept below the species level, and the term isolate when referring to a physical virus from a given variant.

Supplementary figures

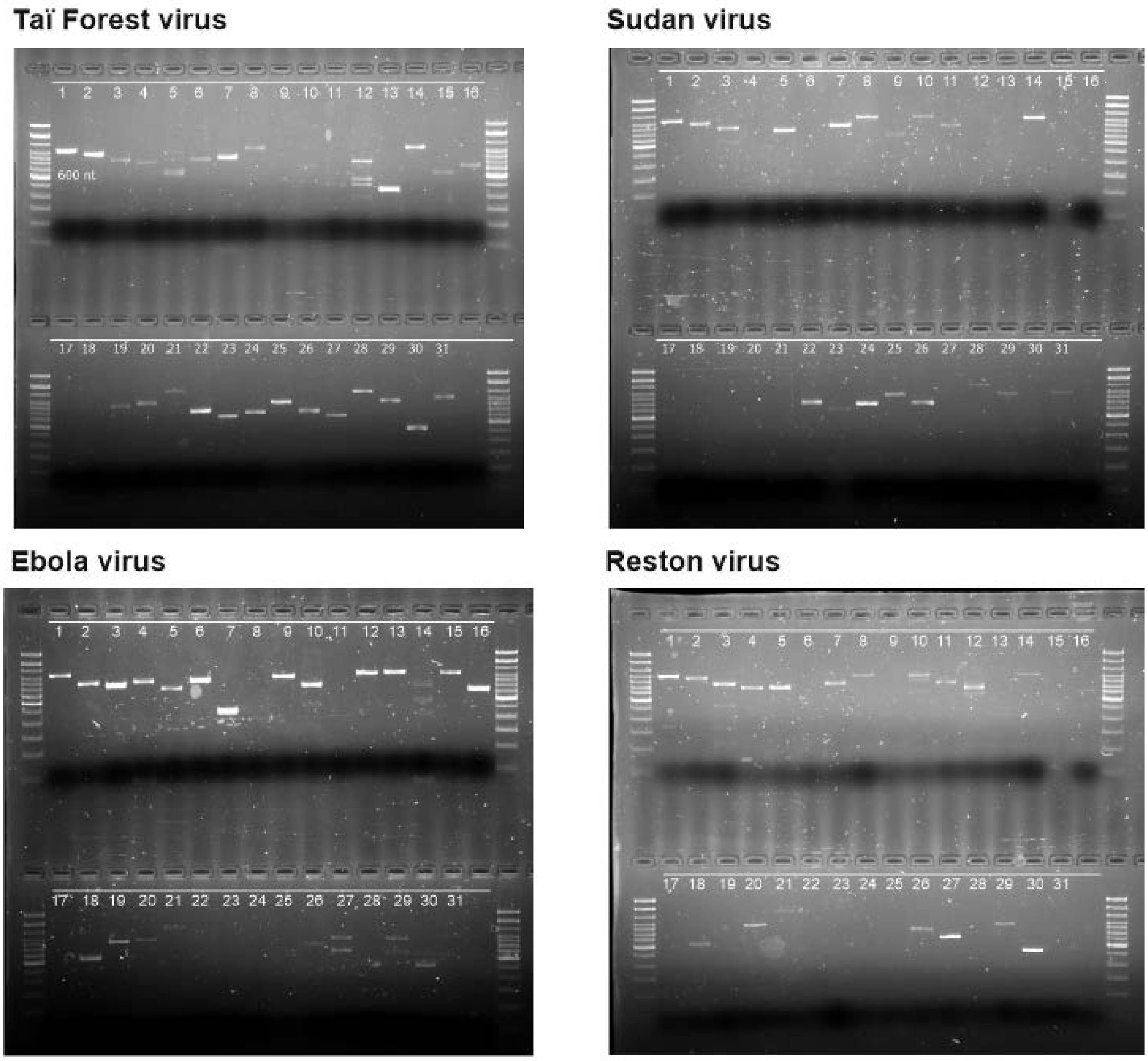

Figure S1. 2% agarose gel images of the generic ebolavirus protocol-derived amplicons corresponding to the 1st round of a reaction containing 10^3 copies. Note: even in the case of faint or unspecific bands, good sequence coverage was obtained during deep sequencing. For less sensitive assays not showing visible amplification in the first round, please see the 2nd round below.

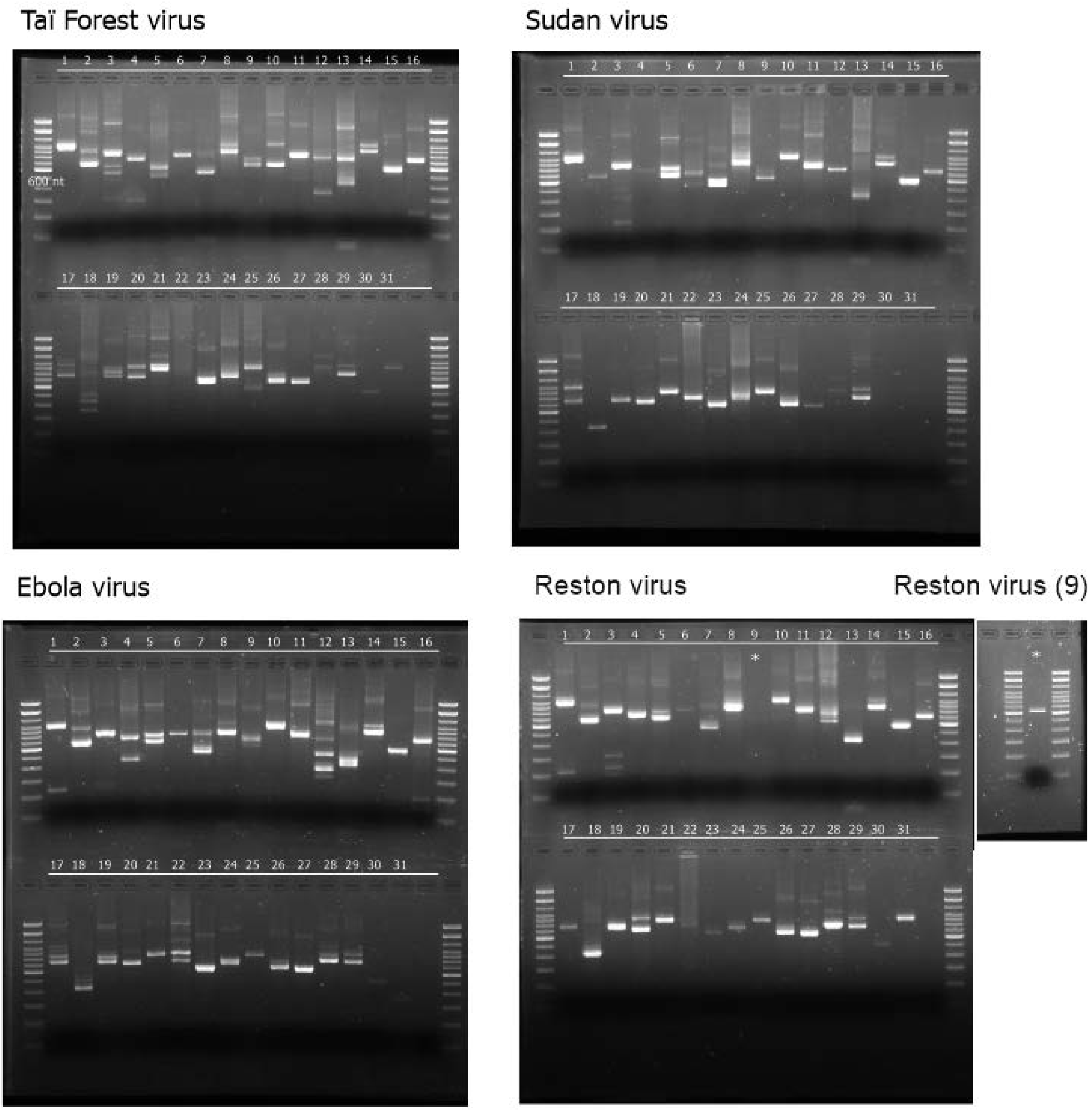

Figure S2. 2% agarose gel images of the generic ebolavirus protocol-derived amplicons corresponding to the 2nd round of a reaction containing 10^3 copies. (*) Amplicon 9 of Reston virus was run separately due to a pipetting error. Note: even in the case of faint or unspecific bands, good sequence coverage was obtained during deep sequencing.

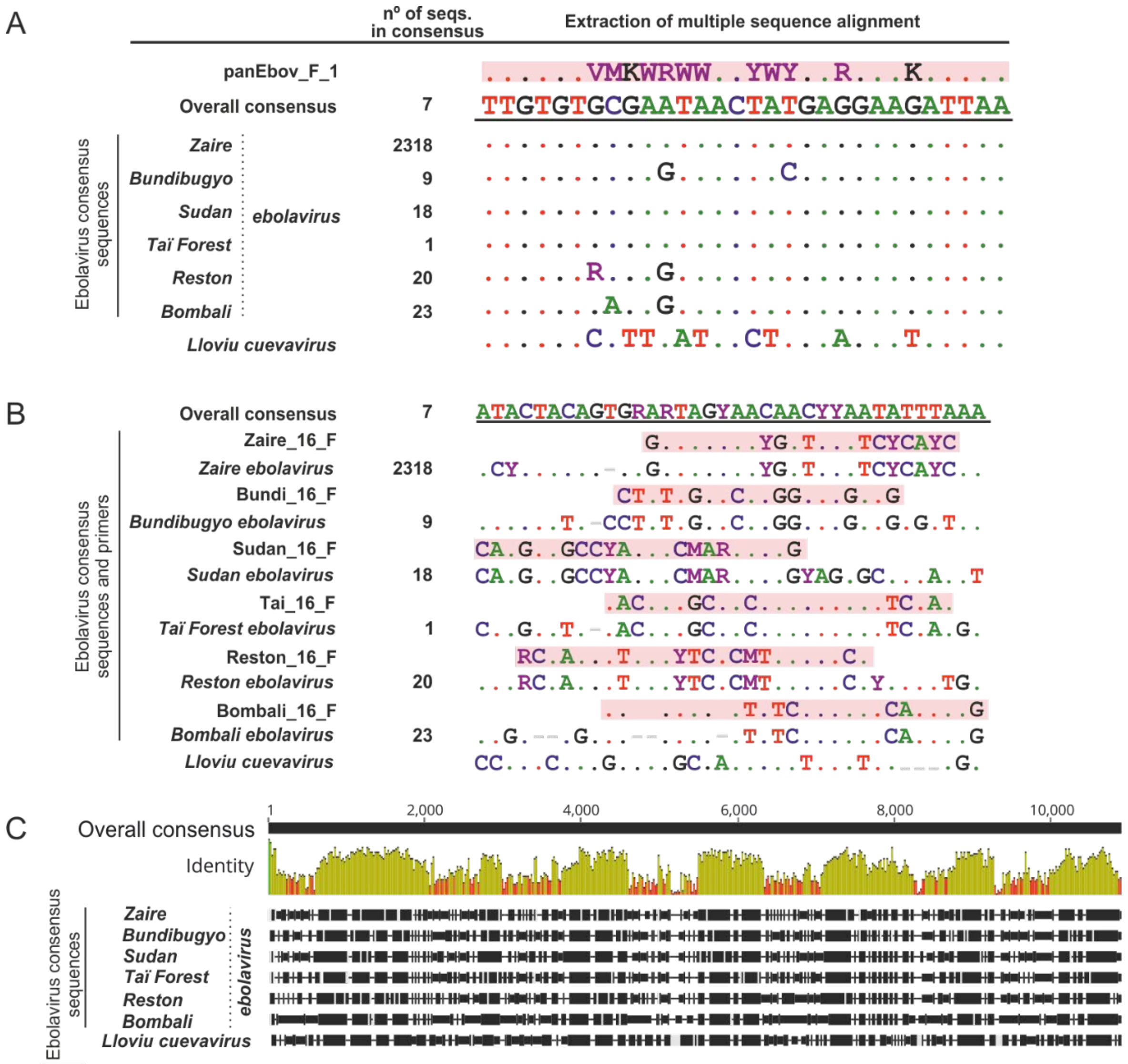

Figure S3. Extraction of the alignment used for protocol design. A. Example of a single degenerated primer working for all viruses in a given region. Consensus sequences of all ebolavirus species are shown together with the number of sequences included from GenBank; Primers are shaded in pink. The overall consensus indicates the consensus of all individual consensus sequences. Lloviu virus included in the design is also shown. B. Example of a genomic region with high diversity between members of individual ebolavirus species where it was not possible to design a single primer and therefore, separate primers to be used in parallel were designed; C. Scheme showing overall identity of genomic sequences of members of the *Ebolavirus* and *Cuevavirus* genera, green areas are conserved, red areas are not.

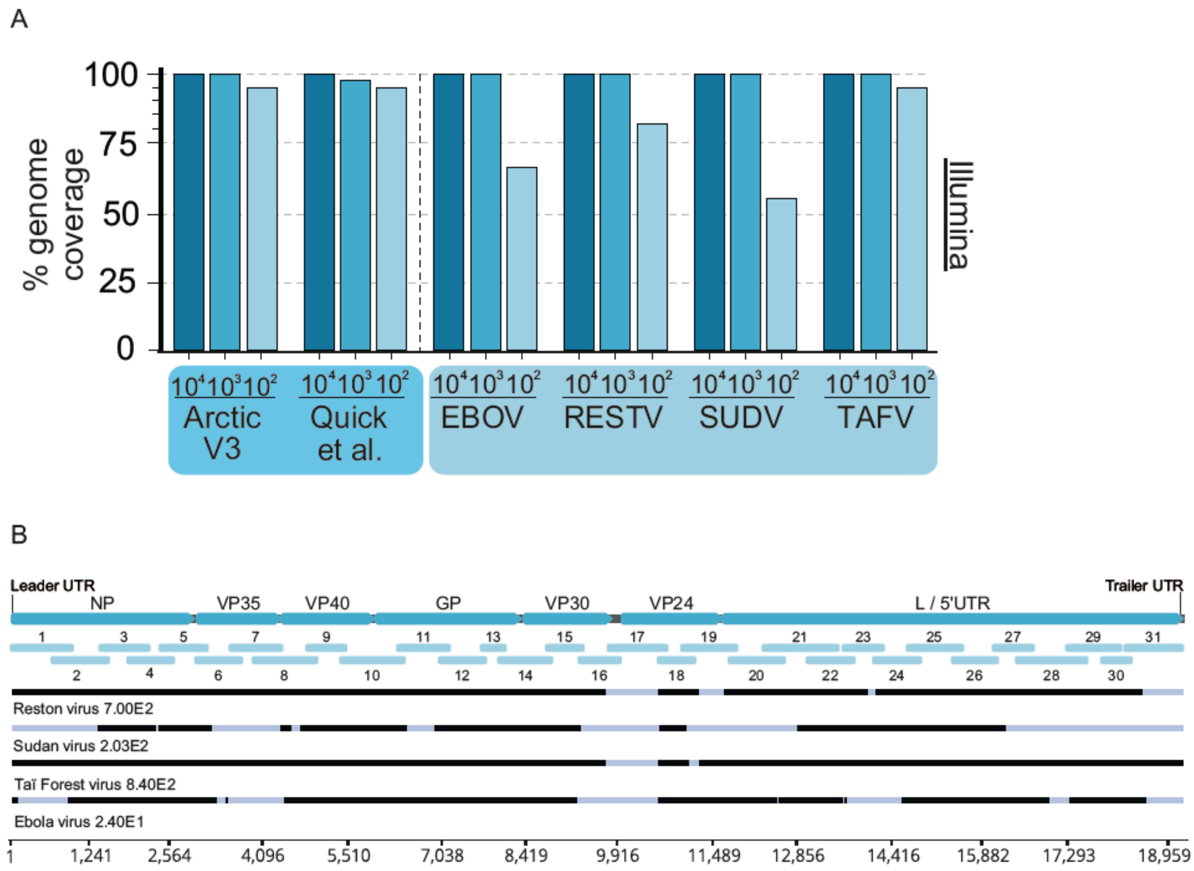

Figure S4. Genome coverage. A. Percentage of genome coverage achieved by each assay with an average of 10^4, 10^3 and 10^2 copies per reaction. B. Schematic representation of the genome coverage based on Illumina MiSeq sequencing at 10^2 copies per reaction. Black lines represent coverage, shaded areas in gray represent lack of coverage. UTR, untranslated region; EBOV: Ebola virus; RESTV: Reston virus, SUDV: Sudan virus; TAFV: Taï Forest virus.

References

1. Katoh K, Misawa K, Kuma Ki, Miyata T. MAFFT: a novel method for rapid multiple sequence alignment based on fast Fourier transform. Nucleic acids research. 2002;30(14):3059-66.
2. Kuhn JH. Guide to the correct use of filoviral nomenclature. Marburg-and Ebolaviruses: Springer; 2017. p. 447-60.

Table S1. Primer scheme, first round. For high throughput on 96-well plates, mix the forward and reverse primers of each assay in 1:1 proportions (i.e., mix all forward primers in one tube and all reverse primers in a second tube) making sure they belong to the same assay and round. In case you prefer to prepare individual master mixes per PCR assay instead, this is also possible. Note that in a few cases, the assay is hemi-nested. Primer working solutions are always 10 µM. Note that the assays are not optimized for multiplexing and should be run in separate wells. **For primer pool preparation examples, see below the table**.

Primer name	Assay n°	Rnd	Sequence (5'-3')	Species covered	Genomic region
1.1_3UTR/NP_F	1.1	1	TTGTGTGMGARTAACTAYGAGGAAIATTAA	All	3' UTR, NP
1.1_3UTR/NP_F-a	1.1	1	TGTGMGARTAACTAYGAGGAAIATTAA	All	
1.1_3UTR/NP_R	1.1	1	CIGAARATNACCATCATRTGYCC	All	
2.1_NP_F	2.1	1	TCTDCATCATGCITAYCARGGDGA	All	NP
2.1_NP_R	2.1	1	GATACCATIGCIITTGTYTGCTG	All	
3.1_NP_F	3.1	1	GCACNCTIGCWGGDGTNAATGT	All	NP
3.1_NP_R	3.1	1	CTGTACDGGWGCNGGWGG	All	
4.1_NP_F	4.1	1	GATGACTTIGTYCTNTTYGATYTDGA	All but Bombali	NP
4.1_NP_F-Bom	4.1	1	GATGACTTGGTACTCTTCGATCT	Bombali	
4.1_NP_R	4.1	1	TIACTIRTGRTGYTGVAGGATWGC	All but Sudan	
4.1_NP_R-Sud	4.1	1	CTCAGTCATGTTGAAGAACRGC	Sudan	
5.1_NP/VP-35_F	5.1	1	AGAWCWCARGGICCWTTTGAIGC	All	NP, VP-35
5.1_NP/VP-35_R	5.1	1	TTTICCIGTCATIAGYTGYTCDGA	All	
6.1_VP-35_F	6.1	1	ATNATTAAGAAAAAAHWHATGATGAAGATT	All	VP-35
6.1_VP-35_F-Sud	6.1	1	TCTTAGCCTAGACRCTGCC	Sudan	
6.1_VP-35_R-Sud	6.1	1	TGATGAAARGCTGTCCCAAAYCC	Sudan	
6.1_VP-35_R	6.1	1	TGGTGRAAHGCWGTICCRAANCC	All	
7.1_VP-35_F	7.1	1	GCMAAATAYGATYTHCTRGTIATGAC	All	VP-35
7.1_VP-35_F-a	7.1	1	GGHCGIGCAACWGCVACYGC	All	
7.1_VP-35_R	7.1	1	VAGGHNISTTTTTCTTAATCTTCATCA	All	
8.1_VP-35-40_F	8.1	1	GGWGACTCYCCBCARTGTGC	All	VP-35, VP-40
8.1_VP-35-40_F-a	8.1	1	GGHAGMACRAACTCYTGVARGAA	All	
8.1_VP-35-40_R	8.1	1	TGIACWGGHGGHAGMACRAACTC	All	
9.1_VP-40_F	9.1	1	TTYATMYTIGARGCWATIGTSAATGT	All	VP-40
9.1_VP-40_R	9.1	1	GGAAGACTIGCHGGRGARTGRCA	All but Sudan	
9.1_VP-40_F-Sud	9.1	1	AGCAACTGGCRGGTGAATGACA	Sudan	
10.1_VP-40/GP_F	10.1	1	YAARAATGGHCARCCHATNATYCC	All but Bombali	VP-40, GP
10.1_VP-40/GP_F-Bom	10.1	1	GAACGGTCAGTCGATCATACC	Bombali	
10.1_VP-40/GP_R	10.1	1	GGTTGTAGCARTTYTCHGCCCA	All	
11.1_GP_F	11.1	1	GRCTIAATCTNGARGGIARYGGA	All	GP
11.1_GP_R	11.1	1	CCTTGACTGTGCANTIGAACCA	Ebola, Bombali	
11.1_GP_F-a	11.1	1	CTTGAIGTYTYGCANTIGAACCA	Taï, Bundibugyo	
11.1_GP_R-a	11.1	1	TTCTGSTRTGWGCANTIRAACCA	Sudan, Reston	

12.1_GP_F	12.1	1	GIGAGTGGGCYTTYTGGGAAA	All	GP
12.1_GP_R	12.1	1	SCCYTCHGTGTADATICCYTC	All	
13.1_GP_F	13.1	1	GCAATCCHAATYTDCAYTAYTGGAC	All	
13.1_GP_R	13.1	1	CCDATWCCIGCHGGDAYCCA	All	
14.1_GP/VP-30_F	14.1	1	TGTATHGARCCHCAWGATTGGAC	All	GP, VP-30
14.1_GP/VP-30_R	14.1	1	TGYTIGAARTCMTCWGCHRTIGGAT	All	
15.1_VP-30_F-a	15.1	1	GTYCCSAMTITSTTTCATCGGAA	Taï, Bundibugyo, Reston	VP-30
15.1_VP-30_F-b	15.1	1	GHGTHCCTAYIGTNTTYCATAAGAA	Ebola, Sudan, Bombali	
15.1_VP-30_R	15.1	1	GADATWACGACWGHIGARCTYTC	All but Sudan	
15.1_VP-30_Sud	15.1	1	ATATCACTACRGTGGAGCTCTC	Sudan	
16.1_VP-30-24_F	16.1	1	GTTTTGARGCWGCMYTRTGGC	All	VP-30, VP-24
16.1_VP-30-24_R	16.1	1	TIRGACMTYYICATTAATCTTCATC	All but Bombali and Reston	
16.1_VP-30-24_R-Res	16.1	1	ATTCCTCCGCAATTAATCTTCATC	Reston	
16.1_VP-30-24_R-Bom	16.1	1	TTGAAAGTCGGTATTAATCTTCATC	Bombali	
17.1_VP-30-24_F-Zai	17.1	1	GGTAGCAAYGATCCATCYCAYC	Ebola	VP-30, VP-24
17.1_VP-30-24_F-Tai	17.1	1	TACAATGCCACCAACTTAATTCTAA	Taï	
17.1_VP-30-24_F-Bom	17.1	1	GGTAGATCTCCCCAATCATTAAG	Bombali	
17.1_VP-30-24_F-Sud	17.1	1	CAAGTAGCCYAGAACMARAACAG	Sudan	
17.1_VP-30-24_F-Bun	17.1	1	CTATTGGTCACGGCTCGATG	Bundibugyo	
17.1_VP-30-24_R	17.1	1	CAAAGTTAAAYTCTCMTAACCTCA	Reston	
17.1_VP-30-24_R	17.1	1	GTIBYIGTWGTIAGHARCCAATC	All but Bombali	
17.1_VP-30-24_R-Bom	17.1	1	GTGCTTGTCACTAATAACCAATC	Bombali	
18.1_VP-24_F	18.1	1	MTTGGGCWGGIMTYGARTTTGA	All	VP-24
18.1_VP-24_R	18.1	1	ATIGCIAGVGAGCTRTTRAAYTC	All	
19.1_VP-24/L_F	19.1	1	TGCICTNCATGTYGTIAAYTACAA	All	VP-24, L
19.1_VP-24/L_R	19.1	1	GTAYTINARTCIRTATATGTTTYGG	All	
19.1_VP-24/L_Zai	19.1	1	TATTTYAAACGGTAIATATGTTTCGG	Ebola	
20.1_L_F	20.1	1	GCTACHCARCATACVCARTAYCC	All	L
20.1_L_R	20.1	1	TTWTTIGAGCAIARTTGDATYTTDGC	All	
21.1_L_F	21.1	1	CCITATGGNGAYTAYRTTTTYTGGA	All	L
21.1_L_R	21.1	1	TCACIYTCBGTNACWACCATCAT	All	
22.1_L_F	22.1	1	AGTCARGGNWCITGGTAYAGTGT	All	L
22.1_L_R	22.1	1	ATGNGGIGGRTTATARTAATCRCT	All	
23.1_L_F	23.1	1	CVTCITGGCAYCAYACHAGTGA	All	L
23.1_L_R	23.1	1	CHAGIGTHCCYTGNAGRTCATC	All	
24.1_L_F	24.1	1	AGYTTRGCMAAAGTHACVAGTGC	All	L
24.1_L_R	24.1	1	CTIGGHGTICGIGARAADATATC	All	
25.1_L_F	25.1	1	GGAAYTGTASTGCVATTGAYTTTGT	All	L
25.1_L_R	25.1	1	IATGNACRATRTTICCIGARTAATG	All	

26.1_L_F	26.1	1	ACAGAGGATAARATHGGDCARCC	All	L
26.1_L_R	26.1	1	TGRGTDGTGAAIGAYCIIGTTTC	All	
27.1_L_F	27.1	1	TICCICAYTTDTCHGGDTGGGA	All	L
27.1_L_R	27.1	1	TCCAGTGCYARIGAYGCATG	All	
28.1_L_F	28.1	1	GTGATMGAGGRYTNTCDGATGC	All	L
28.1_L_R	28.1	1	TTYTCTGTHGTYTCDGCATCCAT	All	
29.1_L_F	29.1	1	ACIGGIRTWGTHTCITCMATGCA	All	L
29.1_L_R	29.1	1	AAGTGRTGTTTGRGTBCGACT	All	
30.1_L/5UTR_F-a	30.1	1	AGTSCHAGRTCWAGTGAGTGGTA	Taï, Sudan, Ebola	L, 5' UTR
30.1_L/5UTR_F-Bun	30.1	1	AGTCCAAAGTCAAGCGAATGGTA	Bundibugyo	
30.1_L/5UTR_F-b	30.1	1	TGCCCGITCWAGTGARTGGTA	Reston, Bombali	
30.1_L/5UTR_R	30.1	1	GTATGATAGAAYCIAITRCAVACA	All	
31.1_L/5UTR_F	31.1	1	GGICGIATHACWAAAYTRGTVAATGA	All	L, 5' UTR
31.1_L/5UTR_R_Zai	31.1	1	GAAGAAATAGATTTATTTTTAAATTTTTGTG	Ebola	
31.1_L/5UTR_R_Sud	31.1	1	GAGAAAATACAGATTTTTTAATTTATTTGTG	Sudan	
31.1_L/5UTR_R_Bom	31.1	1	GAGAAAACAATTTRGTAAAGATATTTTTGTG	Taï, Bundibugyo	
31.1_L/5UTR_R_Res	31.1	1	TATAATGTTTTTGTGTGCGCTTAAC	Reston	
31.1_L/5UTR_R_Bom	31.1	1	GGAAAAAACAGATGACTATTTTTGTG	Bombali	

Abbreviations: Rnd: Round; Inosine (I), R (A or G), Y (C or T), S (G or C), W (A or T), K (G or T), M (A or C), B (C or G or T), D (A or G or T), H (A or C or T), V (A or C or G); Reston (Res), Tai (Taï), Sudan (Sud), Ebola (Zai), Bundibugyo (Bun), Bombali (Bom).

Primer pool preparation examples:

- Assay 1.1:
  - The forward primer mix contains **0.5 µL** of 1.1_3UTR/NP_**F** + **0.5 µL** of 1.1_3UTR/NP_**F**-a that compose the 1 µL added to the mastermix or plate in the bench protocol below.
  - There is only one reverse primer in this assay, so use **1 µL** of 1.1_3UTR/NP_**R**.
- Assay 31.1:
  - There is only one forward primer in this assay, so use **1 µL** of 31.1_L/5UTR_**F**
  - There is five reverse primers in this assay, so use **0.2 µL** of 31.1_L/5UTR_**R**_Zai + **0.2 µL** 31.1_L/5UTR_**R**_Sud + **0.2 µL** 31.1_L/5UTR_**R**_Bom + **0.2 µL** 31.1_L/5UTR_**R**_Res + **0.2 µL** 31.1_L/5UTR_**R**_Bun that compose the 1 µL added to the mastermix or plate in the bench protocol below.

Total amount is always 2 µL containing 1 µL of forward and 1 µL of reverse primers per reaction or per well (primers are always at 10 µM).

Table S2. Primer scheme, second round. For high throughput on 96-well plates, mix the forward and reverse primers of each assay in 1:1 proportions (i.e., mix all forward primers in one tube and all reverse primers in a second tube) making sure they belong to the same assay and round. In case you prefer to prepare individual master mixes per PCR assay instead, this is also possible. Note that in a few cases, the assay is hemi-nested. Primer working solutions are always 10 µM. Note that the assays are not optimized for multiplexing and should be run in separate wells. **For primer pool preparation examples, see below the table**.

Primer name	Assay nº	Rnd	Sequence (5'-3')	Species covered	Genomic region
1.1_3UTR/NP_F	1.1	2	TTGTGTGMGARTAACTAYGAGGAAIATTAA	All	NP, 3' UTR
1.1_3UTR/NP_F	1.1	2	TGTGMGARTAACTAYGAGGAAIATTAA	All	
1.1_3UTR/NP_F	1.2	2	CATCATGTGTCCHACYGAYTGCCA	All	
2.2_NP_F	2.2	2	AAAARGCBTGYCTIGARAARGTICA	All	NP
2.2_NP_R	2.2	2	GCTGATTTCRTTYTTYTIYTGRTGGA	All	
3.2_NP_F	3.2	2	TAAATGTIGGDGARCARTAYCARCA	All	NP
3.2_NP_F-a	3.2	2	CTGCCACTGARGCTGARAARCA	All	
3.2_NP_R	3.2	2	TACIGGDGCNGGWGGDGC	All	
4.2_NP_F	4.2	2	CTITTYGATYTIGAVGAIGABGABGA	All but Bombali	NP
4.2_NP_F-Bom	4.2	2	CTCTTCGATCTGGACGACAATGA	Bombali	
4.2_NP_R	4.2	2	TTRTTYYIGWGRTTCATIACNGGCCA	All	
5.2_NP-VP35_F	5.2	2	CTTGAAGRIGMVTATCCDCCVTGG	All	NP, VP-35
5.2_NP-VP35_F-a	5.2	2	CCDCCVTGGYTVASTGARAARGA	All	
5.2_NP-VP35_R	5.2	2	IGTCATIARYTGCTCDGARAWCCA	All	
6.2_VP-35_Zai	6.2	2	CTAAYGATGAAGATTAAAAACCTTCATC	Ebola	VP-35
6.2_VP-35_F-Bom	6.2	2	AAGAATCTGATGAAGATTAAGGCCC	Bombali	
6.2_VP-35_F-Sud	6.2	2	GGATARGACTTATAGACATCATGGA	Sudan	
6.2_VP-35_F	6.2	2	ATAYWTGATGAAGATTAAAACCTTCATC	Sudan, Taï, Bundibugyo, Reston	
6.2_VP-35_R-Sud	6.2	2	CCGTTCGGATCTTTCAATTTAGC	Sudan	
6.2_VP-35_R	6.2	2	TCCAAADCCWGGIAGRTGRTCATA	All	
7.2_VP-35_F	7.2	2	GCIGCWACWGARGCITAYTGG	All	VP-35
7.2_VP-35_R	7.2	2	TTTIAGICCVAGIGTYTTHCCRTC	All	
8.2_VP-35-40_F	8.2	2	ACCMAARATIGAIMGDGGYTGGGT	All	VP-35, VP-40
8.2_VP-35-40_R	8.2	2	AGIAGYCIBAGIGGRTGATCVGG	All	
9.2_VP-40_F	9.2	2	TTYATMYTIGARGCWATIGTSAATGT	All	VP-40
9.2_VP-40_F-Sud	9.2	2	AGCAACTGGCRGGTGAATGACA	Sudan	
9.2_VP-40_R	9.2	2	TCACARTCYIGWGTIATBACCAT	All	
10.2_VP-40/GP_F	10.2	2	CCTGTTCTIYTICCAAARTAYATYGG	All	VP-40, GP
10.2_VP-40/GP_R	10.2	2	TCAGCCCATTCHCCNGCTTC	All	
11.2_GP_F	11.2	2	ATCIGCRACIAARMGHTGGGG	All	GP
11.2_GP_R	11.2	2	TGSAGIGGRATYTTYTGDARCCA	All	

12.2_GP_F	12.2	2	GARTGGGCYTTYTGGGAAAMTAAAA	All	GP
12.2_GP_R	12.2	2	CIGGYCCGAARTAHGGIATCCA	All	
13.1_GP/VP-30_F	13.1	2	GCAATCCHAATYTDCAYTAYTGGAC	All	GP, VP-30
13.2_GP/VP-30_R	13.2	2	CCAYTGTYTCCAICCHGTCCA	All	
14.2_VP-30_F	14.2	2	CMCATGATTGGACHAARAAYATHAC	All	VP-30
14.2_VP-30_R	14.2	2	TCTTTTTTGCARAATYIRCTRTCRCA	All	
15.1_VP-30-24_R	15.1	2	GADATWACGACWGHIGARCTYTC	All but Sudan	VP-30, VP-24
15.1_VP-30-24_R-Sud	15.1	2	ATATCACTACRGTGGAGCTCTC	Sudan	
15.2_VP-30-24_F	15.2	2	WGTTCCICCRGCACCHAARGA	All	
16.1_VP-30-24_R	16.1	2	TIRGACMTYYICATTAATCTTCATC	All but Bombali and Reston	VP-30, VP-24
16.1_VP-30-24_R-Res	16.1	2	ATTCCTCCGCAATTAATCTTCATC	Reston	
16.1_VP-30-24_R-Bom	16.1	2	TTGAAAGTCGGTATTAATCTTCATC	Bombali	
16.2_VP-30-24_F	16.2	2	CWGCMYTATGGCARCARTGGGA	All	
17.2_VP-24_F-BunTai	17.2	2	ATGATGAAGATTAATGCGGAGGTC	Bundibugyo, Taï	VP-24
17.2_VP-24_F-Zai	17.2	2	GACCTCCGCATTAATCTTCATCAT	Ebola	
17.2_VP-24_F-Res	17.2	2	AGGATGAAGATTAATTGCGGAGG	Reston	
17.2_VP-24_F-Sud	17.2	2	GATGAAAATTAATGAGAAGGTTCC	Sudan	
17.2_VP-24_F-Bom	17.2	2	CGATGAAGATTAATACCGACTTTC	Bombali	
17.2_VP-24_R	17.2	2	CMAGRATIACYCIYARWGCCCA	All but Bombali	
17.2_VP-24_R-Res	17.2	2	CAAAATYACCCTCARAGCCCAG	Reston	
17.2_VP-24_R-Sud	17.2	2	GCAAGAATTACCCTYAARGCCCA	Sudan	
18.2_VP-24/L_F	18.2	2	TTTGCYCCWGCMTGGICRATGAC	All	VP-24, L
18.2_VP-24/L_R	18.2	2	TGGCHGATTTRTCDGGYTCYTG	All	
19.2_L_F	19.2	2	TCATMATIACYMGIACWAAYATGGG	All but Bundibugyo	L
19.2_L_F-Bun	19.2	2	ATCATCACAAGAACCAACATGGG	Bundibugyo	
19.2_L_R	19.2	2	CAWGCWCIDGTIACHAGRTCACA	All	
20.2_L_F	20.2	2	CAATATCCRGAYGCWAGNTTRTC	All	L
20.2_L_R	20.2	2	GACAIGTIAYIADRTCYTTRCACAT	All	
21.2_L_F	21.2	2	CCWCATGCWICHAHIGAYTGGTA	All	L
21.2_L_R	21.2	2	TTRGGYTCGAAIACWGCRTCCCA	All	
22.2_L_F	22.2	2	TTWTTCTCIACSAARRTIATTAGYGA	All	L
22.2_L_F-Zai	22.2	2	AAGGATCTTGGTACAGTGTTAC	Ebola	
22.2_L_R-Zai	22.2	2	TCACTGACATGCATATAACACTG	Ebola	
22.2_L_R	22.2	2	ATAATAATCRCTIACATGYATRTAACA	All	
23.2_L_F	23.2	2	AGTGATGATTTYGGWGARMATGC	All	L
23.2_L_R	23.2	2	GIAGRTCATCAAADATWGCATCDGA	All	
24.2_L_F	24.2	2	GATGAGACITTYGTNCAYTCAGG	All	L
24.2_L_F-Zai	24.2	2	GCCTAGCAAAAGTTACAAGTGC	Ebola	
24.2_L_R	24.2	2	TATCGGCIGCRAAVCIRCTCAT	All	

25.2_L_F	25.2	2	TTAAATGTNCCIGGVTCRCARGA	All	L
25.2_L_R	25.2	2	GAGTAATGWGADGGIGTCATTTG	All	
26.2_L_F	26.2	2	GITTIACHTGGGTHACWCAAGG	All	L
26.2_L_R	26.2	2	TTTCHCCRCTGCTRATIGGRTC	All	
27.2_L_F	27.2	2	TCIGGITGGGARYTAGCNAARAC	All	L
27.2_L_R	27.2	2	TGGAAGAAATTRCTIGCWGTRCT	All	
28.2_L_F	28.2	2	GTGATMGAGGRYTNTCDGATGC	All	L
28.2_L_R	28.2	2	TTYTCTGTHGTYTCDGCATCCAT	All	
28.2_L_F-a	28.2	2	GTCCCTTTATGGATAGTATATCC	Ebola, Reston	
28.2_L_F-b	28.2	2	ACTCCICTRTGGGTHRTMTACCC	All but Ebola and Reston	
28.2_L_R-b	28.2	2	CCIGMVCCYTCWCCYTCIGC	All	
29.2_L_F	29.2	2	TCITCMATGCATTATAARYTDGATGA	All	L
29.2_L_R	29.2	2	ACYTTYTCYAAIGAWGGRAAACC	All	
30.2_L/5UTR_F	30.2	2	AGTSCHAGRTCWAGTGAGTGGTA	Taï, Sudan, Ebola	L, 5' UTR
30.2_L/5UTR_F-Bun	30.2	2	AGTCCAAAGTCAAGCGAATGGTA	Bundibugyo	
30.2_L/5UTR_F-a	30.2	2	TGCCCGITCWAGTGARTGGTA	Reston, Bombali	
30.2_L/5UTR_R	30.2	2	GTATGATAGAAYCIAITRCAVACA	All	
31.2_L/5UTR_F	31.2	2	GGICGIATHACWAAAYTRGTVAATGA	All	L, 5' UTR
31.2_L/5UTR_F-Res	31.2	2	AATCACAAARTTRGTCAATGACTTTC	Reston	
31.2_L/5UTR_R-Zai	31.2	2	TGATGTATGAGAGCAATTTATRAGT	Ebola	
31.2_L/5UTR_R-Tai	31.2	2	GACATCGTGATCGAGCTTTCC	Taï	
31.2_L/5UTR_R-Res	31.2	2	GCGCTTAACATTCTTAKAATGTTTGCAT	Reston	

Abbreviations: Rnd: Round; Inosine (I), R (A or G), Y (C or T), S (G or C), W (A or T), K (G or T), M (A or C), B (C or G or T), D (A or G or T), H (A or C or T), V (A or C or G); Reston (Res), Tai (Taï), Sudan (Sud), Ebola (Zai), Bundibugyo (Bun), Bombali (Bom).

Primer pool preparation examples:

- Assay 24.2:
  - The forward primer mix contains **0.5 µL** 24.2_L_**F** + of **0.5 µL** of 24.2_L_**F**_Zai.
  - There is only one reverse primer in this assay, so use **1 µL** of 24.2_L_**R**.
- Assay 30.2:
  - The forward primer mix contains three primers, so use **0.33 µL** of 30.2_L/5UTR_F + **0.33 µL** of 30.2_L/5UTR_F-Bundibugyo + **0.33 µL** of 30.2_L/5UTR_F-a.
  - The reverse mix contains only one primer, so use **1 µL** of 30.2_L/5UTR_R.

Total amount is always 2 µL containing 1 µL of forward and 1 µL of reverse primers per reaction or per well (primers are always at 10 µM).

Professor Jan Felix Drexler
Charité – Universitätsmedizin Berlin
Institute of Virology
e-mail: felix.drexler@charite.de

DZIF German Center for Infection Research

CHARITÉ UNIVERSITÄTSMEDIZIN BERLIN

Generic ebolavirus assay bench protocol

Updated on 22nd January 2020, from Postigo-Hidalgo et al., Pre-emptive genomic surveillance of emerging ebolaviruses, Eurosurveillance (2020)

Part 1: cDNA synthesis

1. Example formulation using Thermo Fisher Super Script III reverse transcriptase (RT; other reagents may yield equivalent results, but performance has to be verified under individual laboratory conditions). Prepare two mixes separately as described below and proceed as described in "Mix set":

cDNA synthesis example formulation					
**Mix 1**	1x (µL)	10x (µL)	**Mix 2**	1x (µL)	10x (µL)
H_2O (RNAse free)	9	90	1st Strand Buffer (5x)	2	20
DMSO (5%)	1	10	PCR-grade BSA	0.5	5
Random hexamer primers (100 µM)	0.5	5	DTT	1	10
1st Strand Buffer (5x)	2	20	SS III	0.5	5
dNTPs (10 mM)	1	10	-	-	-
RNA	2.5	25	-	-	-
Subtotal	16	160	Subtotal	4	40
**Total**: 200 µL of cDNA (at least 155 µL will be needed for the 31 1st round PCR assays)					

RT conditions		
Mix set	Temp.	Time.
Place Mix 1 on cycler	65ºC	5'
Cool down Mix 1	Ice block	1'
Return Mix 1 to cycler	22ºC	Hold
Add Mix 2 to Mix 1	22ºC	10'
Joint Mix 1 & 2	42ºC	60'
Joint Mix 1 & 2	70ºC	15'
Cool-down	4ºC	Hold

(' = minutes)

Professor Jan Felix Drexler
Charité – Universitätsmedizin Berlin
Institute of Virology
e-mail: felix.drexler@charite.de

Part 2: Primer preparation and PCR settings

1. Prepare assay pools of forward and reverse primer-pairs as described for each assay in Table S1 and Table S2. Note that the individual PCR assays are not optimized for multiplexing and should be run in separate wells (i.e., do not mix assay 1 with assay 2 and so forth in the same well or tube). In case you prefer to prepare individual master mixes per PCR assay instead, this is also possible, simply ignore the steps on adding primers to wells in a 96-well plate and add primers to individual master mixes instead of following steps 2 and 3.
2. Transfer 2 µL of each primer pool to a 96-well plate and prepare the master-mix as described below for the 1st and 2nd round.
3. Add 23 µL of the prepared mix to each well containing the primer pools. If you prefer to use individual master mixes per assay, pipet the full 25 µL instead.

PCR example formulation (Platinum Taq, Thermo Fisher$)

**1st Round**	1x Rx (µL)	35x Rx (µL)*	**2nd Round**	1x (µL)	x35 (µL)
H_2O (RNAse free)	13.65	477.75	H_2O (RNAse free)	17.6	616
10X PCR Buffer, Minus Mg	2.5	87.5	10X PCR Buffer, Minus Mg	2.5	87.5
$MgCl_2$ (50 mM)	1.3	45.5	$MgCl_2$ (50 mM)	1.3	45.5
Fwd primer 1st (10 µM)	1	Added in plate	Fwd primer 2nd (10 µM)	1	Added in plate
Rev primer 1st (10 µM)	1	Added in plate	Rev primer 2nd (10 µM)	1	Added in plate
dNTPs (10 mM)	0.5	17.5	dNTPs (10 mM)	0.5	17.5
Platinum Taq	0.1	3.5	Platinum Taq	0.1	3.5
cDNA Template	5	175	PCR Product	1	35
Total	23 / 25	-	Total	23 / 25	-

$(other reagents may yield equivalent results, but performance must be verified under individual laboratory conditions)
*Amount calculated for 35 reactions (31 assays + 4 reactions pipetting reserve); Rx: Reaction, Fwd, forward; Rev, reverse

Thermocycling protocol

**1st Round**	Temp.	Time.	Cycles	**2nd Round**	Temp.	Time
Denaturation*	95°C	2'		Denaturation	95°C	2'
Denaturation	95°C	15''	45x	Denaturation	95°C	15''
Annealing	57°C	35''		Annealing	60°C	35''
Extension	70°C	1'		Extension	70°C	1'
Final denat.	70°C	2'		Final denat.	70°C	2'
Cool-down	4°C	Hold		Cool-down	4°C	Hold

(' = minutes; '' = seconds) *Denaturation/activation times may differ depending on the enzyme used.
